# Translation and cross-cultural validation of the Lithuanian version of the sleep-related breathing disorder scale of the Pediatric Sleep Questionnaire

**DOI:** 10.3389/fped.2024.1507404

**Published:** 2025-01-07

**Authors:** Gintare Oboleviciene, Valdone Miseviciene

**Affiliations:** ^1^ Pediatric Department, Medical Academy, Lithuanian University of Health Sciences, Kaunas, Lithuania; ^2^ The Center of Pediatric Chronic Respiratory Diseases, Pediatric Department, Medical Academy, Lithuanian University of Health Sciences, Kaunas, Lithuania

**Keywords:** sleep-disordered breathing, pediatric sleep questionnaire, sleep apnea, screening tool, validation

## Abstract

**Introduction:**

Sleep-disordered breathing (SDB) is considered the second most common chronic health condition in children. Untreated SDB is associated with long-term health consequences. Our objective was to translate the Pediatric Sleep Questionnaire (PSQ) into Lithuanian and culturally adapt and validate the translated version in order to improve the diagnosis of SDB in Lithuanian children.

**Methods:**

Translations and cultural adaptations were performed to generate a Lithuanian version of the PSQ. Psychometric analysis was conducted on 112 Lithuanian children aged 2–17 years. All patients underwent overnight polysomnography.

**Results:**

The Lithuanian PSQ showed good internal consistency (Cronbach's alpha = 0.816). Lithuanian PSQ responses administered 14–30 days apart were strongly correlated (r = 0.924, *p* < 0.001, 95% CI 0.830–0.967). EFA of the Lithuanian PSQ confirmed four factors (“snoring”, “behavior”, “sleepiness”, and “other”). We found a sensitivity of 72.7% and specificity of 64.6% for a Lithuanian PSQ cutoff score of 8 to predict moderate-to-severe apnea, and a sensitivity of 85.0% and specificity of 62.0% to predict severe apnea. Using a Lithuanian PSQ cutoff ratio of 0.4, a sensitivity of 72.7% and specificity of 63.3% were found to predict moderate-to-severe apnea, and a sensitivity of 85.0% and specificity of 60.9% were found to predict severe apnea.

**Conclusions:**

The Lithuanian version of the PSQ is a reliable, validated, and culturally adapted screening tool for the prediction of moderate-to-severe sleep apnea in children aged 2–17 years. However, polysomnography should be performed to confirm the diagnosis of sleep apnea and other SDB, and to determine the degree of the disorder and the need for treatment.

## 1 Introduction

Sleep-disordered breathing (SDB) is characterized by abnormal respiration during sleep and includes snoring, obstructive sleep apnea (OSA), central sleep apnea (CSA), and sleep-related hypoventilation (1, 2). OSA is the most common type of SDB, with a prevalence of up to 13% in the pediatric population (1, 2). CSA is relatively rare, usually asymptomatic in children, and commonly associated with other underlying diseases (3). Nocturnal hypoventilation can be observed with or without apneic events and is usually associated with obesity, chest deformities, and neuromuscular disorders (1, 2, 4). Underdiagnosed and untreated pediatric SDB is associated with various outcomes, including behavioral disturbances, hyperactivity, learning difficulties, growth delays, and possible long-term negative effects on cardiovascular health (2, 5). Therefore, the early diagnosis of SDB is important and can be life changing if addressed during childhood or adolescence. According to the American Academy of Sleep Medicine (AASM), polysomnography (PSG) is recommended when clinical assessment suggests a diagnosis of SDB in children (4). However, its clinical symptoms are often difficult to recognize, particularly in children with comorbidities (2). Furthermore, PSG is expensive and not widely available; therefore, various researchers have suggested questionnaires as the first-line screening tools for children (5, 6). Chervin et al. developed the Pediatric Sleep Questionnaire (PSQ), which aids in the recognition of the most important symptoms and consequences of SDB in children (7). The PSQ performed best among all questionnaires assessing pediatric SDB and showed the highest sensitivity and specificity (5, 7–9). The questionnaire has already been translated and validated in Arabic, Hebrew, French, Spanish and other languages (10–13). Our study aimed to translate the PSQ into Lithuanian and culturally adapt and validate the translated version in a cohort of Lithuanian children.

## 2 Methods

This cross-sectional study was conducted at the Lithuanian University Health Sciences Hospital (LUHSH), Kauno Klinikos Center of Pediatric Chronic Respiratory Diseases. The study was approved by the Kaunas Regional Bioethics Committee (BE-2-34, P1-BE-2-66/2020). Written informed consent was obtained from the parents or legal guardians of the children and adolescents prior to commencement of the study. The PSQ developed by Chervin et al. was used in this study (7), and permission to translate the English version of the PSQ into Lithuanian was granted by its original authors. This questionnaire included 22 closed-ended questions aimed at identifying snoring, difficulty breathing during sleep, daytime sleepiness, inattentive or hyperactive behavior, and other pediatric SDB features. The English PSQ contains three relevant domains: snoring (Q1–4), sleepiness (Q10–13), and behavior (Q17–22). The responses for all items are “Yes” (1), “No” (0), and “Don't know” (missing value). The cumulative score is calculated from the “Yes” and “No” responses only. The questionnaire takes approximately 5 min to complete.

This study was divided in two phases: (1) the translation and cultural adaptation of the PSQ and (2) psychometric analysis using the Lithuanian PSQ.

### 2.1 Phase 1: translation and cultural adaptation of the PSQ

The translation of the English PSQ into Lithuanian was performed according to Sousa and Rojjanasrirat's published guidelines (14). This process included (1) the original English PSQ forward translation into Lithuanian by two independent translators with a medical background; (2) a review of two versions of the translated PSQ by a third independent translator and researchers; (3) the Lithuanian PSQ back-translation into English by bilingual independent translators who had a medical background but were blinded to the original English PSQ; and (4) a comparison of two back-translated versions by all translators and researchers, and reparation of the final version of the Lithuanian PSQ.

### 2.2 Phase 2: psychometric analysis using the Lithuanian PSQ

This study included children aged 2–17 years who were examined at the LUHSH Center of Pediatric Chronic Respiratory Diseases between September 2020 and January 2024. The sample size was determined according to the guidelines of Tsang et al. using a ratio of sample size to the item number in the questionnaire of 5:1 (15). The Lithuanian PSQ was completed by the parents of the participants, who understood Lithuanian perfectly, before the PSG was performed. Twenty-five parents completed the Lithuanian PSQ twice-14–30 days apart, to test the long-term stability.

Clinical data, including medical history, clinical examination results, and anthropometric data, were collected during the study period. Standard overnight PSG (Alice 6 with Sleepware G3, Philips Respironics Inc., Murrysville, Pennsylvania) was performed, and encompassed frontal, central, and occipital electroencephalography (EEG), electrooculography, submental electromyography (EMG), nasal and oral airflow, anterior tibialis EMG, body position, thoracic and abdominal movement, as well as oxygen saturation, capnography, and electrocardiography results. All children had a minimum of 4 h sleep recorded. Sleep staging and the assessment of respiratory events were conducted in accordance with the AASM criteria, by a pediatric pulmonologist with extensive experience in pediatric sleep medicine. A subset (*n* = 12) of the recordings was scored twice by two other experts to determine intra-scorer reliability. Mild apnea was diagnosed if the patient had an apnea-hypopnea index **(**AHI) of 1–5/h, moderate if 5–10/h, and severe if exceeding 10/h (16). Sleep hypoventilation was diagnosed if the nocturnal end-tidal carbon dioxide level (EtCO_2_) or transcutaneous carbon dioxide measurement (TcCO_2_) exceeded 50 mmHg for >25% of the total sleep time (1).

### 2.3 Statistical analysis

Statistical analysis was performed using SPSS software (version 29.0; IBM Corp, Armonk, NY, USA.). Descriptive statistics were used to compute the mean and median values along with confidence intervals for the continuous variables under examination. Cronbach's alpha was used to assess internal consistency. A positive rating for internal consistency was determined when Cronbach's alpha was between 0.70 and 0.95 (17). The long-term stability of the translated questionnaire was determined using Spearman's rank correlation coefficient.

Construct validity was determined using exploratory factor analysis (EFA) of the principal components with orthogonal (quartimax) rotation. Quartimax rotation is used to extract a few factors by gathering as many variables as possible under a single factor (18). Bartlett's test of sphericity was used to indicate that the correlation matrix was not random, and the Kaiser–Meyer–Olkin (KMO) test was applied to measure sampling adequacy, which was required to be above 0.5 (18). A cut-off value of 0.2 in communalities was used to include items for further EFA (19).

Spearman's rank correlation coefficient was also used for the analysis of correlations between the Lithuanian PSQ and PSG indices. Diagnostic accuracy was calculated using the receiver operating characteristic (ROC) method. To determine criterion-referenced standards for the Lithuanian PSQ score cutoff points that identify moderate-to-severe OSA risk, ROC curves were constructed, and the area under the ROC curve was calculated. Sensitivity and specificity were determined using established cutoff points. Statistical significance was set at *p* < 0.05.

## 3 Results

### 3.1 Translation and adaptation of the PSQ

The translation and back-translation processes were performed without irregularities. The researchers and translators found no discrepancies between the original PSQ and the primary version of the Lithuanian PSQ. However, the committee decided that Q21 (Is “on the go” or often acts as if “driven by a motor”) should be changed to the Lithuanian expression meaning “is restless in a place” (*Lithuanian: nenustygsta vietoje).* Also, Q5 was assumed to be potentially unclear for parents and changed from “Has trouble breathing, or struggles to breathe” to “has heavy breathing” (*Lithuanian: sunkiai kvėpuoja*).

### 3.2 Characteristics of the study population

Our study included 112 Lithuanian children aged 2–17 years, of whom 61.6% (*n* = 69) were boys and 38.4% (*n* = 43) were girls. The median age was 10 years (95% CI 9.18–10.91). The native language of all the children was Lithuanian. Obesity was identified in 38.4% of the participants. The characteristics of the study population are summarized in Table 1. Descriptive data for the positive answers to each item of the Lithuanian PSQ are presented in Table 2.

**Table 1:** Characteristics of study population. Variables are presented as frequencies (percentages) and median (95% CI).

Variables	Study population
Sex, males	69 (61.6)
Age, years	10 (9.18–10.91)
Nationality, Lithuanian	112 (100)
BMI	18.8 (20.63–24.68)
Overweigh	5 (4.5)
Obesity	43 (38.4)
Non-respiratory sleep disorders	14 (12.5)
Adenoid hypertrophy	16 (14.3)
Tonsillar hypertrophy	24 (21.4)
Neuromuscular diseases	24 (21.4)
Allergic rhinitis	23 (20.5)
OSA	80 (71.4)
Mild	48 (60.0)
Moderate	12 (15.0)
Severe	20 (25.0)
Sleep hypoventilation syndrome	14 (12.5)

**Table 2:** Descriptive statistics for items of Lithuanian-PSQ.

	Item	Yes,% (*n*)
Q1	Snore more than half the time	20.5 (23)
Q2	Always snore	9.8 (11)
Q3	Snore loudly	39.3 (44)
Q4	Have “heavy” or loud breathing	47.3 (53)
Q5	Have trouble breathing, or struggle to breath *(Lithuanian version: have heavy breathing)*	8.0 (9)
Q6	Stop breathing during the night	33.0 (37)
Q7	Tend to breathe through the mouth during the day	33.0 (37)
Q8	Have a dry mouth on waking up in the morning	41.1 (46)
Q9	Occasionally wet the bed	21.4 (24)
Q10	Wake up feeling unrefreshed in the morning	36.6 (41)
Q11	Have a problem with sleepiness during the day	42.8 (48)
Q12	Has a teacher commented that your child appears sleepy during the day	36.6 (41)
Q13	It is hard to wake your child up in the morning	25.9 (29)
Q14	Does your child wake up with headaches in the morning	16.1 (18)
Q15	Did your child stop growing at a normal rate at any time since birth	20.5 (23)
Q16	Is your child overweight	35.7 (40)
Q17	Does not seem to listen when spoken to directly	38.4 (43)
Q18	Has difficulty organizing tasks and activities	31.3 (35)
Q19	Is easily distracted by extraneous stimuli	50.9 (57)
Q20	Fidgets with hands or feet or squirms in seat	38.4 (43)
Q21	Is “on the go” or often acts as if “driven by a motor” *(Lithuanian version: is restless in a place)*	40.2 (45)
Q22	Interrupts or intrudes on others	37.5 (42)

### 3.3 Internal consistency and test-retest reproducibility

The Lithuanian PSQ showed good internal consistency, with a Cronbach's alpha coefficient of 0.816 for all items. The subscales of snoring (Q1–4), sleepiness (Q8, Q10, Q12, Q15), behavior (Q17–22), and other symptoms (Q9, Q11, Q16) had Cronbach's alpha coefficients of 0.730, 0.559, 0.773, and 0.541, respectively. The long-term stability of the Lithuanian PSQ was evaluated in a sample of 25 participants and showed a statistically significant correlation (r = 0.924, *p* < 0.001, 95% CI 0.830–0.967).

### 3.4 EFA of the Lithuanian PSQ

**Figure 1:**
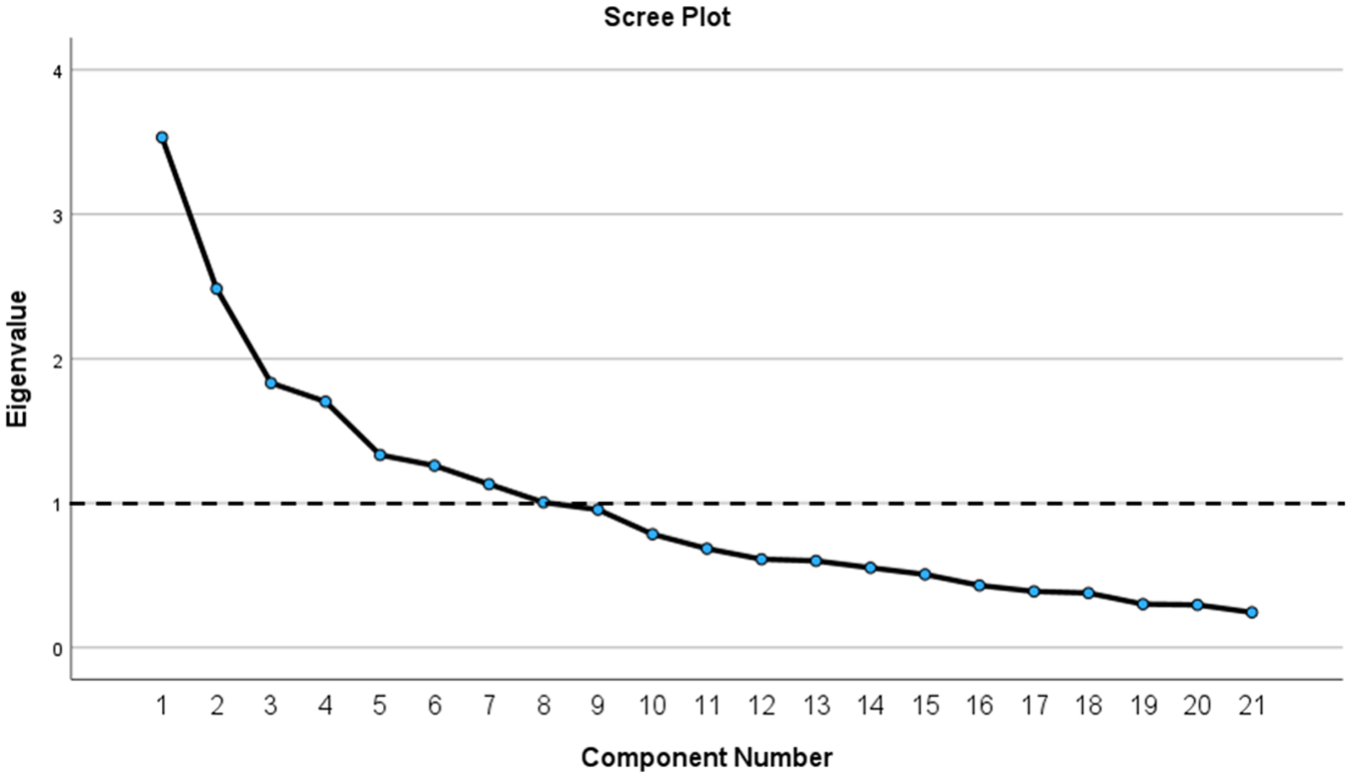
Scree plot obtained from EFA of Lithuanian-PSQ.

The results of Bartlett's test of sphericity indicated that the correlation matrix was not random; *χ*2 = 499.467, df = 210, *p* < 0.001. The KMO index confirmed sampling adequacy with a value of 0.639. Before rotation, factor analysis using the principal components method revealed eight factors with eigenvalues >1, explaining 66.04% of the cumulative variance. To further identify potentially meaningful factors, a scree plot was constructed (Figure 1). Based on the original PSQ by Chervin et al. the number of factors, factors with eigenvalues >1, and eigenvalues that accounted for >5% of the variance, four factors were extracted. Additionally, the four factors explained 43.47% of the variance. All four factors had at least three salient loadings, with values of ≥0.40. Communalities were reasonably strong, ranging from approximately 0.23–0.68, except for Q14, which had a value of 0.084. Q14 was excluded from the factorial analysis. After the rotation, four items (Q5, Q6, Q7, and Q13) failed to load sufficiently on any factor at a minimum of 0.4.

Factors extracted from the Lithuanian PSQ were labelled according to meaningful connections between items and subscales in the original PSQ by Chervin et al.: “snoring,” “behavior,” “sleepiness” and “other” (7). Factors related to “snoring” and “behavior” were identical to those in the original PSQ while “sleepiness” and “other” differed slightly (7). In the Lithuanian PSQ, Q8 and Q15 were allocated to the “sleepiness” domain and Q11 was allocated to the “other” domain. Table 3 presents the factor structures.

**Table 3:** Factor structure of Lithuanian-PSQ [cut off 0.4, items (Q5, Q6, Q7, Q13) without loading > 0.4 were removed; Q14 were excluded from EFA with <0.2 value in communalities].

Lithuanian-PSQ items		Factor 1	Factor 2	Factor 3	Factor 4
		Behavior	Snoring	Sleepiness	Other
Q20	Fidgets with hands or feet or squirms in seat	0.767			
Q22	Interrupts or intrudes on others	0.724			
Q19	Is easily distracted by extraneous stimuli	0.698			
Q17	Does not seem to listen when spoken to directly	0.688			
Q21	Is “on the go” or often acts as if “driven by a motor” *(Lithuanian version: is restless in a place)*	0.635			
Q18	Has difficulty organizing tasks and activities	0.478			
Q1	Snore more than half the time		0.809		
Q2	Always snore		0.785		
Q3	Snore loudly		0.693		
Q4	Have “heavy” or loud breathing		0.444		
Q8	Have a dry mouth on waking up in the morning			0.760	
Q12	Has a teacher commented that your child appears sleepy during the day			0.664	
Q10	Wake up feeling unrefreshed in the morning			0.549	
Q15	Did your child stop growing at a normal rate at any time since birth			0.463	
Q11	Have a problem with sleepiness during the day				0.657
Q16	Is your child overweight				0.621
Q9	Occasionally wet the bed				0.470

### 3.5 Concurrent validity of the Lithuanian PSQ

All patients included in this study underwent PSG. Strong inter-scorer reliability was observed between the AHI of the two assessments (r = 0.925, *p* < 0.001, 95% CI 0.685–0.984). A weak correlation was observed between the AHI and the Lithuanian PSQ score (r = 0.393, *p* < 0.001, 95% CI 0.218–0.543) as well as between the AHI and the Lithuanian PSQ ratio (r = 0.337, *p* < 0.001, 95% CI 0.156–0.496) in the study population.

**Figure 2:**
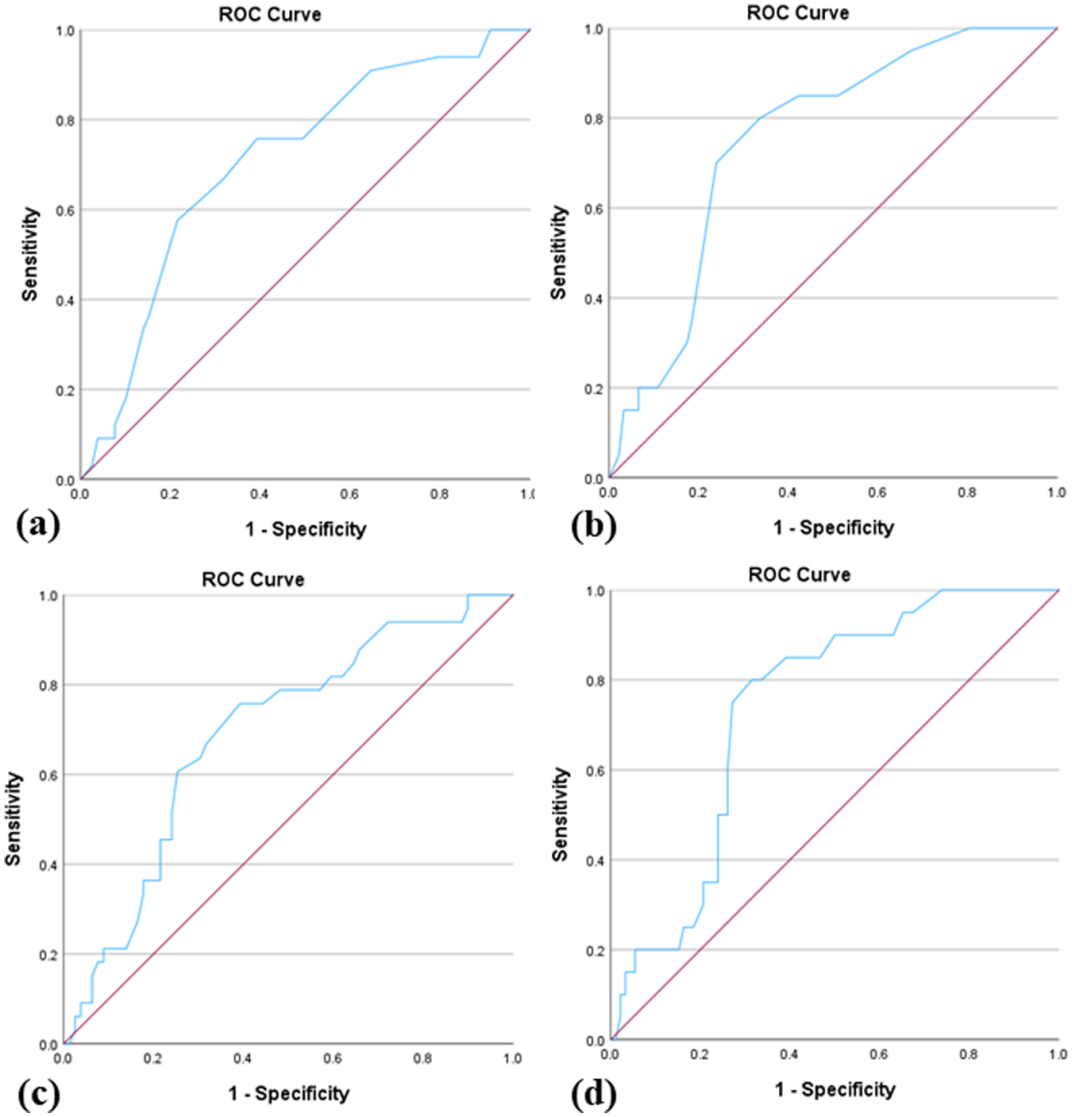
Receiver operating characteristic (ROC) curves: **(a)** ROC curve for prediction of moderate-severe apnea by using Lithuanian-PSQ score; **(b)** ROC curve for prediction of severe apnea by using Lithuanian-PSQ score; **(c)** ROC curve for prediction of moderate-severe apnea by using Lithuanian-PSQ ratio; **(d)** ROC curve for prediction of severe apnea by using Lithuanian-PSQ ratio.

ROC curve analysis was performed to evaluate the validity of the Lithuanian PSQ for the prediction of moderate and severe sleep apnea. Using the Lithuanian PSQ score, the area under the curve (AUC) for the prediction of AHI > 5 was 0.709 ± 0.53 (*p* < 0.001, 95% CI 0.606–0.813) and for the prediction of AHI > 10, 0.752 ± 0.53 (*p* < 0.001, 95% CI 0.648–0.856). The data showed that the optimal Lithuanian PSQ score cutoff value to predict AHI > 5 and AHI > 10 was 8 positive answers. Using the Lithuanian PSQ ratio, the AUC for the prediction of AHI > 5 was 0.691 ± 0.53 (*p* = 0.002, 95% CI 0.586–0.795) and for the prediction of AHI > 10, 0.739 ± 0.52 (*p* = 0.001, 95% CI 0.637–0.841). During the evaluation of the Lithuanian PSQ ratio, the optimal cut-off value to predict AHI > 5 and AHI > 10 was 0.4. All the ROC curves are shown in Figure 2.

Using the ROC method, we observed a sensitivity of 72.7% and specificity of 64.6% for a Lithuanian PSQ cutoff score of 8 to predict moderate-to-severe apnea. A sensitivity of 85.0% and a specificity of 62.0% were observed for the same cutoff score to predict severe apnea. Using a Lithuanian PSQ cutoff ratio of 0.4, a sensitivity of 72.7% and specificity of 63.3% were found to predict moderate-to-severe apnea. A sensitivity of 85.0% and a specificity of 60.9% were found for the same cutoff ratio to predict severe apnea. Their operating characteristics are listed in Table 4.

**Table 4:** Operating characteristics of the Lithuanian-PSQ at cut-off scores for prediction of moderate-severe and severe apnea.

	Sensitivity (95% CI)	Specificity (95% CI)	Kappa	LR+	LR-	AUC (95% CI)	*p*-value
≥8 score for AHI > 5	72.7% (0.558–0.849)	64.6% (0.536–0.742)	0.319	2.052	0.422	0.709 ± 0.53 (0.606–0.813)	<0.001
≥8 score for AHI > 10	85.0% (0.640–0948)	62.0% (0.518–0.712)	0.289	2.234	0.242	0.752 ± 0.53 (0.648–0.856)	<0.001
0.4 ratio for AHI > 5	72.7% (0.558–0.849)	63.3% (0.523–0.731)	0.306	1.981	0.431	0.691 ± 0.53 (0.586–0.795)	<0.001
0.4 ratio for AHI > 10	85.0% (0.640–0.848)	60.9% (0.507–0.702)	0.279	2.172	0.246	0.739 ± 0.52 (0.637–0.841)	<0.001

## 4 Discussion

Sleep-related breathing disorders can occur in children of all ages and are considered the second most common pediatric chronic health conditions (20). Various researchers refer to snoring as the most important nocturnal symptom of obstructive SDB (1, 2, 20, 21). However, the absence of snoring can lead to an underdiagnosis of obstructive SDB in children (21). Other SDB is usually associated with asymptomatic clinical patterns and may be even more unrecognizable, especially in children with complex disorders (2, 3). Fifteen questionnaires were established in order to improve the diagnosis of sleep disorders in children and 4 questionnaires were devoted to the investigation of the risk for SDB: the OSA-18, OSA-5, I'M SLEEPY, and PSQ questionnaires (7, 22–25). The PSQ created by Chervin et al. showed the highest validity (5), therefore we chose to translate, culturally adapt, and validate this questionnaire for the assessment of Lithuanian children.

The Lithuanian PSQ has good internal consistency, with a Cronbach's alpha coefficient 0.816 for all items. This was similar to the original PSQ (Cronbach's alpha = 0.89) and previous studies that translated and validated the PSQ in other languages (Cronbach's alpha ranged from 0.776–0.947) (7, 10–12, 26). In our study, the internal consistency of each subscale was acceptable, with Cronbach's alpha ranging from 0.541–0.773. Our results on the internal consistency of the subscales are consistent with those of Almutairi et al.'s Arabic version and Jordan et al.'s French version of the PSQ (10, 12). It is important to note that various authors used distinct components of subscales that differed from the original version of the PSQ. For example, all questions were used in the factorial analysis of the Hebrew PSQ, while the original PSQ included only 14 items in three subscales (snoring: Q1–4, sleepiness: Q10–13, behavior: Q17–22). The Lithuanian PSQ had different items allocated to the “sleepiness” and “other” subscales and it differed slightly from the original PSQ. Overall, the Lithuanian PSQ showed acceptable internal consistency.

The test-retest reliability of the Lithuanian PSQ, evaluated using Spearman's correlation, was high, suggesting that the scores remained stable over 14–30 days. These results were similar to those of the original PSQ created by Chervin et al. as well as to those of the Arabic and French versions (7, 10, 12, 26). The results of the EFA indicated that the Lithuanian PSQ could be divided into four subscales: “snoring,” “behavior,” “sleepiness,” and “other.” The subscales of “snoring” and “behavior” did not differ from the English version of the PSQ (7). Q8 and Q15 were allocated to the “sleepiness” domain while Q11 and Q13 did not fit statistically into this domain as in the original PSQ (7). However, Q8 and Q15 relate to poor sleep quality and could be fitted to the “sleepiness” subscale. Q11 was allocated to the “other” domain in the Lithuanian PSQ. All other items allocated to this subscale matched the English version of the PSQ (7). It is important to note that five items (Q5, Q6, Q7, Q13, and Q14) were excluded during the EFA process. This exclusion may be attributed to linguistic and cultural specificities, as well as age-related differences. For instance, Q14, which asks about waking up with headaches, might not be suitable for preschool children who often struggle to articulate or localize pain. Other studies on PSQ validation did not perform factorial analyses (10, 13, 27).

The Lithuanian PSQ was validated using the PSG test to determine its concurrent validity and diagnostic accuracy. A relatively weak correlation was observed between the AHI and the Lithuanian PSQ score as well as between the AHI and the Lithuanian PSQ ratio, which highlights a modest level of concurrent validity for the tool. It is important to note that the PSQ primarily captures subjective symptoms of SDB, while the AHI objectively measures apnea and hypopnea events during sleep. The AHI and Lithuanian PSQ had a fair level of agreement according to Cohen's kappa. The findings suggest that while the PSQ is a valuable screening tool, it should not replace PSG for definitive diagnosis. The modest correlation emphasizes the need for clinicians to interpret PSQ results in conjunction with other clinical findings, rather than relying on the tool as a sole indicator of SDB severity. Using the ROC analysis, our findings confirmed that the cutoff value to predict moderate and severe apnea is ≥8 positive answers and it corresponds to the original English version of the PSQ (7). However, a Lithuanian PSQ ratio of >0.4 was set to predict apnea and it differed from the original PSQ (7). Chervin et al. and other authors used a cutoff value of 0.33 in the validation of the Arabic, Hebrew, and Thai versions of the PSQ (7, 10, 11, 27). The elevated number of “don't know” responses in our cohort may explain the slightly higher PSQ ratio threshold. This frequent uncertainty can be attributed to cultural practices in Lithuania, where older children commonly sleep in separate rooms from their parents. Consequently, parents may lack awareness of certain sleep-related symptoms, limiting their ability to provide accurate responses to specific questions. A test with a high proportion of “don't know” answers may be less accurate in predicting sleep apnea, potentially reducing its diagnostic reliability. Notably, we used an AHI threshold of 5, whereas other authors used an AHI threshold of 1 (10, 11, 27). An AHI threshold of 5 was used in the original English version of the PSQ (7). Unfortunately, this study did not validate the Lithuanian PSQ for predicting mild sleep apnea, which limits its applicability in the early detection of the disease. However, the detection of moderate-to-severe apnea is crucial (AHI > 5) because it is less likely to resolve without treatment and should be treated according to recommendations (1, 16). Future studies are needed to combine the PSQ with other early disease biomarkers to improve the prediction of mild sleep apnea and enhance its clinical applicability.

In terms of the sensitivity and specificity of the Lithuanian PSQ, our findings showed modest diagnostic accuracy using the ROC curve method to predict moderate-to-severe apnea in children aged 2–17 years. An acceptable AUC (≥0.70) was observed for the prediction of moderate-to-severe apnea using the Lithuanian PSQ score (17). In our study, a slightly lower AUC was found for the prediction of moderate sleep apnea using the Lithuanian PSQ ratio, although the AUC was still acceptable for the prediction of severe sleep apnea (17).

Chervin et al. reported very good diagnostic accuracy for the original PSQ, with 81% sensitivity and 87% specificity (7). We observed a similar level of sensitivity for the Lithuanian PSQ in diagnosing moderate-to-severe sleep apnea. Our version of the PSQ had a lower level of specificity in our population than the English version of the PSQ, indicating a lower ability to predict that a child does not have significant SDB (7). In addition, lower specificity was found by other authors who researched the diagnostic accuracy of the Arabic and Thai versions of the PSQ with a specificity of 43.5% and 54%, respectively (10, 27). Ferry et al. re-assessed the diagnostic accuracy of the English version of the PSQ and found an even lower specificity (30%) in predicting moderate OSA in children (28). In our study, we included patients with suspected SDB who were referred for pediatric pulmonology consultation. Therefore, the lower level of specificity may have been influenced by the small number of healthy children without respiratory complaints. Additionally, the high prevalence of obesity in the study population may have contributed to the lower specificity, as certain PSQ symptoms could overlap with those caused by obesity-related conditions. Overall, the Lithuanian PSQ is a reliable and valid tool for the detection of suspected SDB in children with sufficiently high sensitivity. However, the Lithuanian PSQ does not have sufficient specificity and should not be used as a standalone diagnostic tool to replace PSG for diagnosing sleep apnea, particularly for ruling out the disease. The PSQ should be regarded as an initial screening tool to identify cases that require further evaluation through PSG, which remains the gold standard for definitive diagnosis.

This study had some limitations. First, it was a single-center study that included various children aged 2–17 years. They were referred for pediatric pulmonology consultations, and some had comorbidities. This may have introduced sampling bias, which could have influenced the lower specificity observed, as the population studied might not fully reflect the broader pediatric population. For this reason, higher specificity of the PSQ could be expected in the general pediatric population. Assessor bias was a consideration, although it is important to note that a subset of the recordings was scored twice by two experts to rule out potential bias. In addition, we did not validate the PSQ in the prediction of mild sleep apnea in children. Future prospective studies with larger cohorts including children without comorbidities are necessary to confirm our results.

## 5 Conclusions

Lithuanian PSQ is a reliable, validated, and culturally adapted screening tool for predicting moderate-to-severe sleep apnea in 2–17-year-old children. We recommend the use of the Lithuanian PSQ in primary care settings, pediatric pulmonology, otorhinolaryngology, or other consultations to detect suspected SDB and recognize children who require further examination. However, PSG should be performed to confirm the diagnosis of sleep apnea and other SDB as well as to specify the degree of the disorder and the need for treatment.

## Data Availability

The original contributions presented in the study are included in the article; further inquiries can be directed to the corresponding author.
